# Impact of MRI/US fusion‐guided prostate biopsy on biopsy‐naïve patients: A single urologist’s experience

**DOI:** 10.1002/bco2.86

**Published:** 2021-05-04

**Authors:** Muammer Altok, Cihan Demirel, Hyunseon C. Kang, Haesun Choi, David John, Irene A. Inguillo, John W. Davis, John F. Ward

**Affiliations:** ^1^ Department of Urology The University of Texas MD Anderson Cancer Center Houston TX USA; ^2^ Department of Abdominal Imaging Division of Diagnostic Imaging The University of Texas MD Anderson Cancer Center Houston TX USA

**Keywords:** fusion biopsy, prostate cancer, prostate cancer screening

## Abstract

**Objectives:**

To report our experience with imaging‐guided targeted prostate biopsy (IGTpBx) for patients undergoing initial prostate biopsy in a clinical setting.

**Materials and methods:**

From July 2014 to February 2020, 305 men who had IGTpBx performed as their first prostate biopsy were enrolled. Two dedicated magnetic resonance imaging (MRI) radiologists segmented at least 1 region of interest (ROI) for each of these men using screening 1.5T MRI images. A single urologist employed the robotic‐assisted Artemis MRI/ultrasonography (US) fusion platform to obtain 2‐3 targeted samples from each ROI and additional random samples from the zones of the prostate outside the ROIs (a total of 12 zonal samples). Biopsy outcomes were categorized based on the Gleason score (GS) grade group (GG) as no cancer, favorable (GG < 3 or GS < 4 + 3), or clinically significant (GG ≥ 3 or GS ≥ 4 + 3) cancer.

**Results:**

The overall cancer detection rate was 75%:31% clinically significant, 44% favorable, and 25% no cancer. These findings triggered active interventions in 176 (58%) patients. A prostate‐specific antigen (PSA) level of 0–4 ng/mL was detected in 39 (66%) of 59 patients (32 favorable, 7 significant), 4–10 ng/mL in 147 (77%) of 190 patients (85 favorable, 62 significant), and 10 ng/mL and over in 44 (80%) of 55 patients (17 favorable, 27 significant).

**Conclusions:**

The tumor detection rate was 75% with IGTpBx in patients without a previous biopsy. In addition, about 42% of detected cancers were deemed clinically significant and led to active interventions. IGTpBx as a patient’s first prostate biopsy improves the detection of clinically significant prostate cancer when compared with historical data for random systematic prostate biopsy.

## 1 INTRODUCTION

Prostate cancer (PCa) is one of the most common cancers in men worldwide.^1^ Men with a high prostate‐specific antigen (PSA) level and/or abnormal digital rectal examination (DRE) are often advised to undergo a prostate biopsy to establish or exclude the diagnosis. Unlike other solid tumors, prostate cancer is detected using random biopsy sampling of the entire organ. The number of cores considered to be optimal for cancer detection has varied with time; the currently accepted technique is 10‐ to 12‐core laterally directed transrectal ultrasound‐guided biopsy (TRUS‐bx).^2^ This approach has inherent shortcomings: (1) a high false‐negative detection rate of 20‐24% and (2) an understaging rate of 50‐80% for clinically significant prostate cancers.^3^, ^4^ Increasing the number of cores increases the detection of insignificant PCa.^5^, ^6^ In the United States, approximately 1.3 million prostate biopsies are performed annually^7^; therefore, the overtreatment and undertreatment rates can have a significant effect, making an accurate diagnosis and avoidance of multiple unnecessary prostate biopsies increasingly important in PCa.

In the last decade, with the development of multiparametric magnetic resonance imaging (mpMRI), the ability to detect tumor has significantly improved. Many studies have shown that mpMRI could be used as a triage test to avoid unnecessary biopsy for insignificant cancer, such as with imaging‐guided targeted prostate biopsy (IGTpBx).^8^, ^9^, ^10^

IGTpBx is currently only recommended for selected patients with a prior negative biopsy and continued clinical suspicion of prostate cancer.^2^, ^11^ A recently published, multicenter, randomized trial by the PRECISION study group has compared IGTpBx with standard prostate biopsy for PCa diagnosis and reported more clinically significant cancer identification with less over‐detection of clinically insignificant cancers and fewer biopsy cores with IGTpBx.

Understanding the impact of IGTpBx using MRI/US fusion technology on biopsy‐naïve patients may have significant implications for PCa screening recommendations and the economics of PCa detection and treatment. We report our center’s experience with MRI/US fusion‐guided prostate biopsy as the initial diagnostic biopsy for patients undergoing prostate cancer screening, along with surgical pathological correlation when applicable.

## 2 MATERIALS AND METHODS

### 2.1 Study design and patient selection

**FIGURE 1:**
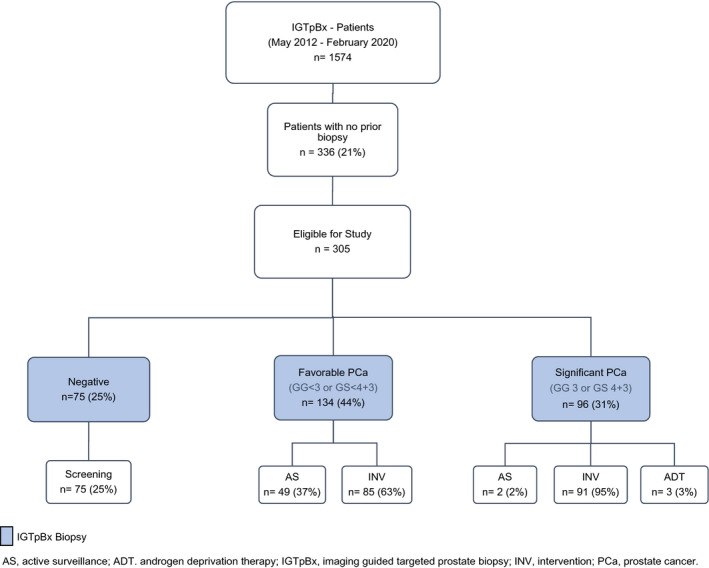
Study diagram. AS, active surveillance; ADT, androgen deprivation therapy; IGTpBx, imaging guided targeted prostate biopsy; INV, intervention; PCa, prostate cancer

Institutional Review Board approval was obtained for this retrospective study (PA16‐0421). From May 2012 through February 2020, IGTpBx was performed in a total of 1574 patients at The University of Texas MD Anderson Cancer Center. Three hundred and thirty‐six (21%) of these patients were biopsy naïve, and IGTpBx was performed as the first prostate biopsy (July 2014–February 2020). After the exclusion of 1 patient with a pathology result of metastasis to the prostate, 1 patient with prior transurethral prostate resection with the diagnosis of PCa, 8 patients whose biopsy was performed by another physician, 15 patients with no region of interest (ROI) biopsy samples obtained, and 6 patients with no random biopsies obtained (by physician decision), the final analysis included 305 patients (Figure 1).

All 305 patients were selected after repeat review by one of two dedicated MRI radiologists who segmented at least 1 ROI as suspicious for cancer using the 5‐point Likert system. This system assigns a score of 1, 2, 3, 4, or 5 to denote the probability of carcinoma as highly unlikely, unlikely, equivocal, likely, or highly likely, respectively.^12^ The utilization of the Likert system scoring reflects our radiologists’ practice. Although PI‐RADS has been recommended by the European Society of Urogenital Radiology and the American College of Radiology, it remains subjective and requires scoring (also on a 5‐point scale) based on the observation of limited sequences (diffusion‐weighted images [DWIs] and T2). The Likert scoring system has been advocated by some expert groups, such as in the United Kingdom.^13^, ^14^ Equivalent or better performance for clinically significant PCa detection has been reported with the Likert system, compared with PI‐RADS, reducing the number of unnecessary prostate biopsies.^15^, ^16^ Although both systems are associated with inter‐observer variability due to subjectivity, the Likert system may allow more freedom to assign a level of suspicion using all imaging sequences available. More importantly, the application of PI‐RADS requires certain technical parameters for high‐quality images (e.g., b‐values of at least 1400 for DWI), which may not be achievable in some institutions (including ours at the time of the data collection).

### 2.2 Biopsy procedure

IGTpBx included 2‐3 targeted samples from each radiographic ROI. An additional single random sample was obtained from regions of the prostate without an ROI for a 12‐zonal prostate biopsy. All biopsies were performed transrectally by a single urologist (Dr. Ward) using the robotic‐guided Artemis MRI/US fusion platform (Eigen, Green Valley, CA, USA) with the patient under monitored anesthetic care without the introduction of local periprostatic blockage. Our protocol for biopsy included a Fleet enema the night before, a neomycin enema the morning of the biopsy, and 1 g of ceftriaxone or 5 mg/kg gentamicin intravenously intraoperatively. Infection rates were under 1% and there were no hospital admissions for sepsis. The most common complication was urinary retention, which occurred in about 5% of patients.

Biopsy results were classified based on Gleason score (GS) grade groups (GG) on a scale ranging from 1 to 5; GG1 (low risk) = GS ≤ 6, GG2 (intermediate favorable) = GS 3 + 4 = 7, GG3 (intermediate unfavorable) = GS 4 + 3 = 7, GG4 (high) = GS 8, and GG5 (high) = GS 9‐10.^17^ Outcomes were categorized as no cancer, favorable cancer (GG < 3 or GS < 4 + 3), or clinically significant cancer (GG ≥ 3 or GS ≥ 4 + 3).^17^

### 2.3 MRI technique

Patients underwent imaging on a 1.5 Tesla or 3 Tesla GE HealthCare Signa HDx MR scanner (GE Healthcare, Waukesha, WI, USA) or a Siemens MR scanner (Siemens, Erlangen, Germany) using an 8‐channel abdominal array coil and, for the majority of patients using an endorectal coil (MR Innerva; Medrad, Pittsburgh, PA, USA). The scanning protocol included small field‐of‐view sagittal, axial, and coronal fast spin‐echo T2‐weighted images, DWIs with apparent diffusion coefficient reconstruction, and dynamic contrast‐enhanced images (DCEs), as well as whole pelvis T1‐weighted images and DWIs with apparent diffusion coefficient reconstruction. DCE MRI was performed after intravenous injection of gadopentetate dimeglumine (Gadavist; Bayer HealthCare Pharmaceuticals, Berlin, Germany) at 0.1 mmol/kg of body weight at a rate of 3 mL/s via a power injector. The images were acquired with a temporal resolution of 11‐14 seconds.

### 2.4 Statistical analysis

Statistical analysis was performed using the SPSS 24.0 software program for Windows (SPSS Inc., Chicago, IL, USA). Descriptive statistics for the clinical, pathological, and treatment‐related data were provided. Pearson’s Chi‐square test (or Fisher’s exact test) was used to analyze categorical variables. Statistical significance was considered as *P* < .05.

## 3 RESULTS

Patient characteristics are provided in Table 1. IGTpBx detected tumors in 230 (75%) of the 305 patients, 96 (31%) of which were clinically significant (Table 2, Figure 1). The biopsy results triggered active interventions in 176 (58%) patients: robot‐assisted radical prostatectomy (RARP) was recommended, planned, or performed in 104; external‐beam radiotherapy in 43; and brachytherapy or cryoablation in 17 patients. In 12 patients, one of these interventions was offered and is still pending for the patient’s decision.

**TABLE 1:** Patient and biopsy characteristics

No. of patients	305
Age (years)	
Median (IQR)	66 (60‐71)
Ethnicity, *n* (%)	
Caucasian	234 (76.7)
African American	24 (7.9)
Asian	14 (4.6)
Hispanic	15 (4.9)
Other	18 (5.9)
PSA (ng/mL)	
Median (IQR)	5.7 (4.4‐8.2)
DRE status, *n* (%)	
Normal	288 (94.4)
Abnormal	17 (5.6)
Likert score (MRI), *n* (%)	
Likert 1‐2	13 (4.3)
Likert 3	67 (22.0)
Likert 4	81 (26.6)
Likert 5	144 (47.2)
Prostate biopsy, *n* (IQR)	
Random	9 (7‐10)
Targeted	4 (3‐6)
Total	13 (12‐14)
ROI	2 (1‐3)

Abbreviations: DRE, digital rectal examination; IQR, interquartile range; MRI, magnetic resonance imaging; PSA, prostate‐specific antigen; ROI, region of interest.

**TABLE 2:** Prostate cancer detection on ROIs and random biopsies

PCa detection, *n* (%)	All	Favorable		Significant
		GG 1	GG 2	GG ≥ 3
Overall	230 (75.4)	43 (14.1)	91 (29.8)	96 (31.5)
Cancer in random biopsies	167 (54.8)	56 (18.4)	59 (19.3)	52 (17.1)
Cancer in random biopsies only	20 (6.6)	11 (3.6)	7 (2.3)	2 (0.7)
Cancer in targeted (ROI) biopsies	210 (68.9)	39 (12.8)	86 (28.2)	85 (27.9)
Cancer in targeted (ROI) biopsies only	63 (20.7)	23 (7.5)	20 (6.6)	20 (6.6)

Abbreviations: GG, Gleason group; PCa, prostate cancer; ROI, region of interest.

In 59 patients with PSA levels of 0 to 4 ng/mL, 39 (66%) had tumors (32 favorable, 7 significant); in 190 patients with PSA of 4 to 10 ng/mL, 147 (77%) had tumors (85 favorable, 62 significant); in 55 patients with PSA greater than 10 ng/mL, 44 (80%) had tumors (17 favorable, 27 significant). One patient whose PSA was unreliable was excluded from this analysis.

Among the 230 patients, tumors were identified within the ROIs from IGTpBx in 210 (91%) patients and from random biopsy in 167 (73%). In 63 (27%) of 230 patients, tumors were detected only in ROIs from IGTpBx [clinically significant in 20 (32%)] and in 20 (9%) only in random biopsies [clinically significant in 2 (10%)] (Table 2).

In this study, IGTpBx in biopsy‐naïve men missed 12% (11 of 96) of the significant cancers that were detected in random samples only (no or favorable cancer in ROIs but significant cancer in random biopsies), whereas random biopsy samples failed to detect 46% (44 of 96) of the significant cancers identified only by ROIs via IGTpBx. In another perspective, random biopsy failed to detect nearly half (44 of 96) of the significant cancers detected in this study.

MRI Likert score correlation with cancer detection also was investigated. Likert 1‐2 scores were reported in only 13 patients. These patients were removed from the analysis to avoid any confusion. The final analysis of MRI Likert scores included only Likert 3‐5 lesions. Details are provided in Table 3. Of 144 patients with Likert 5 lesions, PCa was detected in 135 (94%) of them. Half (50.4%) of these cancers were significant. In Likert 4 and Likert 3 lesions, PCa was detected in 57 (70%) of 81 and 32 (48%) of 67, with 40% and 16% of them being significant cancer, respectively. Compared with Likert 4 and Likert 3 lesions, Likert 5 lesions had significantly higher rates of overall (both *P* < .001) and significant (*P* = .006 and *P* < .001) cancer detection, respectively. Compared with Likert 3 lesions, Likert 4 lesions had significantly higher rates of overall (*P* = .005) and significant (*P* = .001) cancer detection. Of the 2 patients with significant cancer detected by random biopsy only and no cancer on ROI, 1 had a Likert 4 lesion, and the other had a Likert 3 lesion on MRI.

**TABLE 3:** Prostate cancer detection rates based on MRI likert scores

MRI likert score	No. of patients	PCa detection, n (%)			
		Overall	Favorable		Significant
			GG 1	GG 2	GG ≥ 3
Likert 1‐2	13	6 (46.2)	4 (30.8)	2 (15.4)	0 (0)
Likert 3	67	32 (47.8)	10 (14.9)	17 (25.4)	5 (7.5)
Likert 4	81	57 (70.4)	16 (19.8)	18 (22.2)	23 (28.4)
Likert 5	144	135 (93.7)	13 (9.0)	54 (37.5)	68 (47.2)

Abbreviations: GG, Gleason group; GS, Gleason score; PCa, prostate cancer.

RARP was performed in 96 patients. The pathology results for 3 patients were not available (2 patients underwent RARP at another institution and the GS of 1 patient could not be determined due to neoadjuvant hormone therapy). Of the 93 RARP results, 59 (63.4%) of 93 in targeted (ROIs) and 31 (33.3%) of 93 in random biopsies were consistent with the GG/GS results. When the results were grouped as favorable or significant cancer, 78 (83.9%) of 93 targeted biopsies and 59 (63.4%) of 93 random biopsies were consistent with the RARP results. Cancers detected by RARP were reported as no cancer in 3 (3.2%) of 93 targeted (ROIs) and 17 (18.3%) of 93 random biopsies. Significant cancer was detected in 42 RARP specimens; 5 of them (11.9%) were missed in targeted (ROI) biopsies, whereas random biopsy missed in 22 patients (52.4%). Details are provided in Table 4.

**TABLE 4:** Comparison of biopsy results with RARP pathologies

Biopsy types	RARP pathology results	
	Favorable (GG < 3 or GS < 4 + 3)	Significant (GG ≥ 3 or GS ≥ 4+3)
Targeted Biopsy (ROIs) results		
Negative	2 (66.7)	1 (33.3)
Favorable (GG < 3 or GS < 4+3)	41 (91.1)	4 (8.9)
Significant (GG ≥ 3 or GS ≥ 4 + 3)	8 (17.8)	37 (82.2)
Random Biopsy (ROIs) results		
Negative	7 (41.2)	10 (58.2)
Favorable (GG < 3 or GS < 4+3)	39 (76.5)	12 (23.5)
Significant (GG ≥ 3 or GS ≥ 4+3)	5 (20)	20 (80)
Targeted + Random Biopsy (ROIs) results		
Favorable (GG < 3 or GS<4+3)	41 (93.2)	3 (6.8)
Significant (GG ≥ 3 or GS ≥ 4+3)	10 (20.4)	39 (79.6)

Abbreviations: GG, Gleason group; GS, Gleason score; RARP, Robot‐assisted radical prostatectomy; ROI, region of interest.

## 4 DISCUSSION

The main goal of prostate biopsy is to make an accurate diagnosis by detecting significant cancers while minimizing the false‐negative rate and insignificant cancer detection. For many years, TRUS‐bx has been the standard of care. The development of mpMRI enhanced the visualization of the prostate, adding the option of IGTpBx as an alternative to TRUS‐bx, especially in patients who have a targetable suspicious lesion, which comprises at least approximately 60% of biopsy‐naïve patients.^18^

The PROMIS (PROstate MR Imaging Study) was designed to evaluate the diagnostic accuracy of mpMRI and TRUS‐bx against the gold standard of transperineal template prostate mapping (TPM) biopsy.^8^ A total of 576 men underwent a 12‐core systematic TRUS‐bx followed by a TPM biopsy under general anesthesia. Clinically significant cancer was defined as GS ≥ 7 or any grade of cancer ≥4 mm. The results of PROMIS showed that mpMRI has a sensitivity of 93% for the detection of clinically significant prostate cancer with a negative predictive value of 89%. This compared favorably with TRUS‐bx, which had a sensitivity of 48% and a negative predictive value of 74% in that study. MpMRI missed 17 clinically significant cancers, whereas TRUS‐bx missed 119 significant cancers.

However, the results of IGTpBx studies in the literature have been controversial. Mischinger et al. reported no difference in both overall and significant cancer detection rates between targeted and systematic transperineal prostate biopsy with MRI‐TRUS fusion in 130 prostate biopsy‐naïve and 72 prior‐negative‐biopsy patients with targetable lesions on MRI.^19^ Targeted biopsy missed 17% of the clinically significant cancers in that study. Hakozaki et al. reported higher overall cancer (49.7% vs. 58.7%) and significant cancer (48% vs. 57.1%, *P* = .088) detection with standard biopsy compared with MRI/US fusion biopsy in 177 patients with a suspicious (mostly single) lesion on MRI.^20^ MRI/US fusion biopsy missed 22% of the clinically significant cancers whereas standard biopsy missed 10% of them.

Recently, a multicenter, randomized trial was published by the PRECISION study group, which compared IGTpBx biopsy obtained only from ROIs (maximum 4) with standard 10‐ to 12‐core systematic TRUS biopsy for prostate cancer diagnosis in biopsy‐naïve men. They reported that IGTpBx with fewer biopsy cores resulted in more overall (68% vs. 51%) and clinically significant (GS ≥ 7; 55% vs. 27%, *P* < .001) cancer identification with less over‐detection of clinically insignificant cancer.^21^ The design of this study was controversial, however. Two separate cohorts were compared—only patients with PI‐RADS 3‐5 lesions received biopsy in the IGTpBx group while others were excluded, but in the standard biopsy group, MRI was not performed and all patients underwent prostate biopsy. Additionally, this study was not homogenous in terms of operator experience, the usage of an endorectal coil in MRI, and various techniques of IGTpBx with visual registration or software‐assisted registration with either a transrectal or transperineal access route. Another important issue with this study was the possibility of missing clinically significant cancer by the omission of standard biopsy cores in the IGTpBx group. In our study, 20 (9%) of 230 cancers were detected in only standard cores and 9 (45%) of 20 were GS ≥7 (2 of 20 were significant cancer). The percentage of significant cancer that is detected in TRUS‐bx but missed in IGTpBx in the literature varies from 0% to 10%.^22^, ^23^, ^24^, ^25^

In another study conducted by Maxeiner et al., 318 biopsy‐naïve patients underwent real‐time MRI/US fusion‐guided targeted biopsy combined with a TRUS‐guided 10‐core standard biopsy.^26^ The overall cancer detection rate was 77% (245/318). IGTpBx alone detected 67% of the prostate cancers and standard biopsy alone 70%. The combination of IGTpBx and TRUS‐bx detected 195 (61%) clinically significant cancers in 318 patients. IGTpBx alone detected clinically significant cancers in 163 patients (51%) and missed 32 (16%) of them. TRUS‐bx alone detected 145 (46%) clinically significant cancers.

In a meta‐analysis conducted by Schoots, it is shown that the detection of both overall prostate cancers and clinically significant cancers increased with the usage of IGTpBx.^9^

These findings are consistent with our results. The overall cancer detection rate was 75.4% in our study. In the literature, the overall cancer detection rate of standard TRUS‐bx varies from 25% to 45%.^4^, ^27^ In a previous study, we detected PCa in 731 (34.9%) of 2095 patients with standard TRUS‐bx (biopsy scheme ≥10 cores).^28^ Different criteria have been applied to define significant cancer in the literature; therefore, the rate of significant cancer detection varies from study to study. Based on our criteria, the significant cancer detection rate was 32% (96 of 305) and comprised 42% (96 of 230) of the detected cancers in our study (Figure 1, Table 2). If we apply the criterion of GS ≥7 that is used in most studies, the significant cancer detection rate increases to 61% (187 of 305) and comprises 81% (187 of 230) of detected cancers (Table 2). In a newly published study by Ahdoot et al. in *The New England Journal of Medicine*, the same significant disease criteria used in our study were applied, and a combination of standard and MRI‐targeted fusion biopsy was evaluated for PCa diagnosis in 2103 patients.^29^ Overall cancer and significant cancer detection rates were reported as 62.4% and 22.2%, respectively. However, about 80% of these patients were not biopsy naïve and had undergone at least one biopsy before. Targeted biopsy missed ~9% (41 of 466) of the significant cancers that were detected by random biopsy in this study, which is similar to our results.

This high rate of cancer detection with IGTpBx is probably related to two factors: (1) performing targeted biopsy rather than random biopsy and (2) performing the biopsy in a specific group with MRI‐defined possible‐ or probable‐malignancy lesions in the prostate (Likert 3‐5 lesions). An important area of discussion is whether we can omit using random or systemic biopsy and only take biopsies from the lesions detected on MRI. Our results show that IGTpBx alone missed about 12% (11 of 96) of the significant cancers (GG ≥ 3 prostate cancers). Therefore, obtaining random samples should not be omitted and IGTpBx has to be integrated with random prostate biopsy.

The retrospective design and lack of comparison with TRUS‐bx are the main limitations of our study. Another limitation was the number of patients. More patients are needed to evaluate the correlation of biopsy findings with Likert scores and RARP specimens more appropriately. Despite these limitations, the limited number of studies in the literature about the diagnosis of prostate cancer using IGTpBx and the details provided by our study increase its value.

## 5 CONCLUSION

Compared with the commonly cited positive predictive value of an extended random systematic prostate biopsy of about 30%, we found a significantly higher cancer detection rate of 75% when IGTpBx is performed. Nearly half of the detected cancers (42%) were clinically significant and 58% led to active interventions. IGTpBx as a patient’s first prostate biopsy improves the detection of overall and clinically significant prostate cancer when compared with historically reported rates for random systematic prostate biopsy, especially in patients with Likert 4‐5 lesions.

## CONFLICT OF INTEREST

The authors declare that they have no confict of interest.
